# Giant right atrial tumor following catheter ablation

**DOI:** 10.1186/s44215-024-00145-7

**Published:** 2024-02-27

**Authors:** Takeshi Wada, Hirotsugu Hamamoto, Shinji Miyamoto

**Affiliations:** 1 Department of Cardiovascular Surgery, Almeida Memorial Hospital, 1509-2, Miyazaki, Oita, Oita 870-1195 Japan; 2 Department of Cardiovascular Surgery, Oita University Hospital, Oita, Japan

**Keywords:** Cardiac tumor, Myxoma, Right atrial tumor, Angiosarcoma, Catheter ablation

## Abstract

**Background:**

We report a rare case of a giant right atrial myxoma after catheter ablation.

**Case presentation:**

A 74-year-old man presented with a fever of unknown origin three years after laser catheter ablation. Multimodal imaging revealed a giant tumor located in the right atrium, which was suspected to be malignant. Surgical resection was performed, and pathological examination revealed that the tumor was a myxoma.

**Conclusions:**

Several cardiac myxoma cases after catheter ablation have been reported, suggesting a potential association between myxoma development and catheter ablation-related tissue injury.

## Background

Myxomas are the most common benign cardiac tumors; however, a definitive diagnosis requires surgery and pathological examination. Limited information is available regarding myxoma development after catheter ablation. Herein, we present a case of a giant myxoma arising from the right atrium after catheter ablation.

## Case presentation

**Fig. 1:**
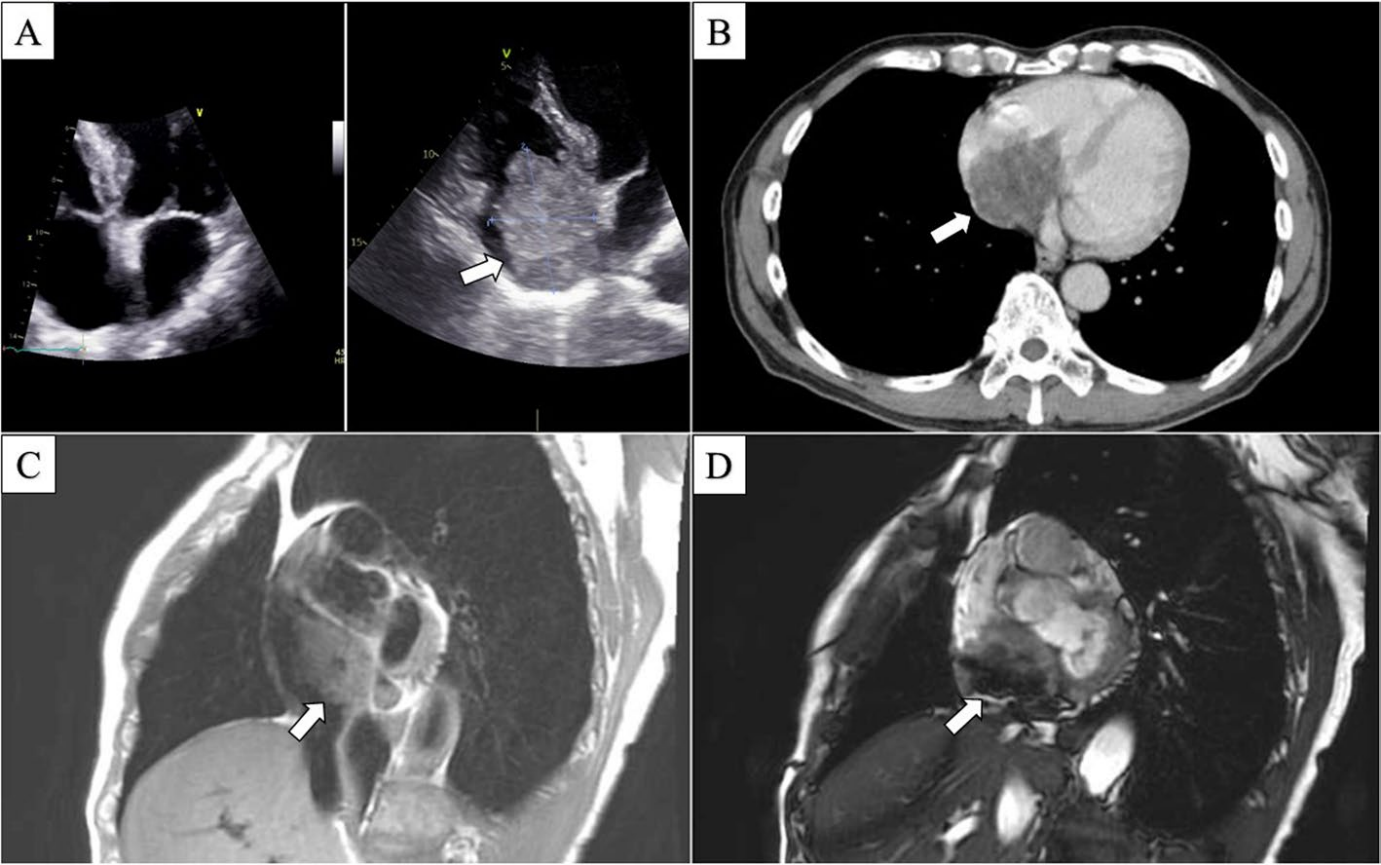
Preoperative images. **A** Transthoracic echocardiogram at the right after laser catheter ablation and the time of hospitalization this time. The latter demonstrates a giant mass in the right atrium (arrow). **B** Contrast-enhanced CT reveals the tumor has an irregular surface, heterogeneous contrast-enhanced effect, and vascular structures within. **C** Cardiac magnetic resonance imaging (CMR) exhibits heterogeneous high T1 signal. **D** CMR reveals a low-to-high T2 signal

A 74-year-old man with elevated C-reactive protein (CRP) levels and a giant right atrial tumor was admitted to our hospital. Five months before admission, the patient had a fever of unknown origin. Transthoracic echocardiography revealed a giant right atrial tumor measuring 63 mm (Fig. 1A) along with mild tricuspid valve regurgitation. The tumor appeared to originate from the atrial septum and moved slightly toward the right ventricle. Contrast-enhanced computed tomography (CT) revealed a tumor with surface irregularities accompanied by heterogeneous contrast effects and vascular structures within the tumor (Fig. 1B). Cardiovascular magnetic resonance imaging (CMR) revealed a heterogeneously high T1 signal and a low-to-high T2 signal (Fig. 1C, D). Three years before admission, the patient had undergone laser catheter ablation for paroxysmal atrial fibrillation, and a transthoracic echocardiography conducted during that period revealed the absence of a tumor. These findings suggested the presence of a rapidly growing giant tumor in the right atrium, characterized by hemorrhagic necrosis and significant blood vessel proliferation, raising the suspicion of angiosarcoma.

**Fig. 2:**
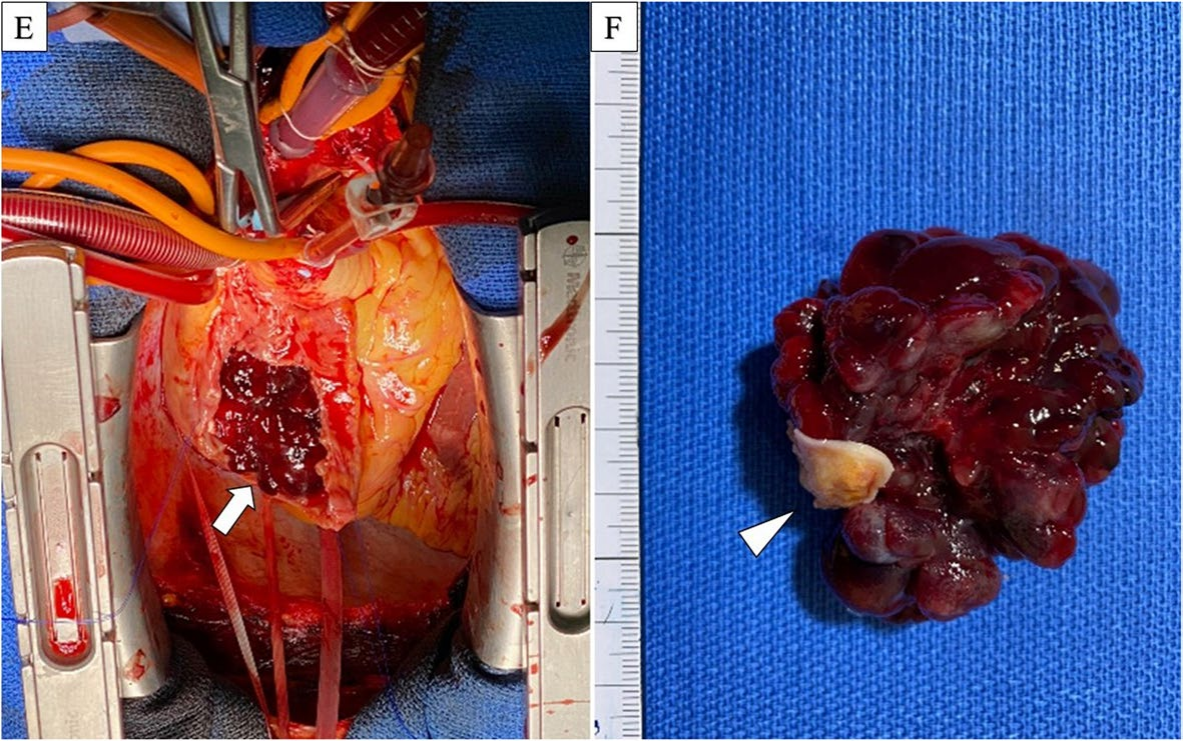
Intraoperative images. **E** The arrow indicates the tumor in the right atrium. **F** The tumor was dark red, grape cluster-like, papillary. The arrowhead demonstrates the normal right atrium tissue

**Fig. 3:**
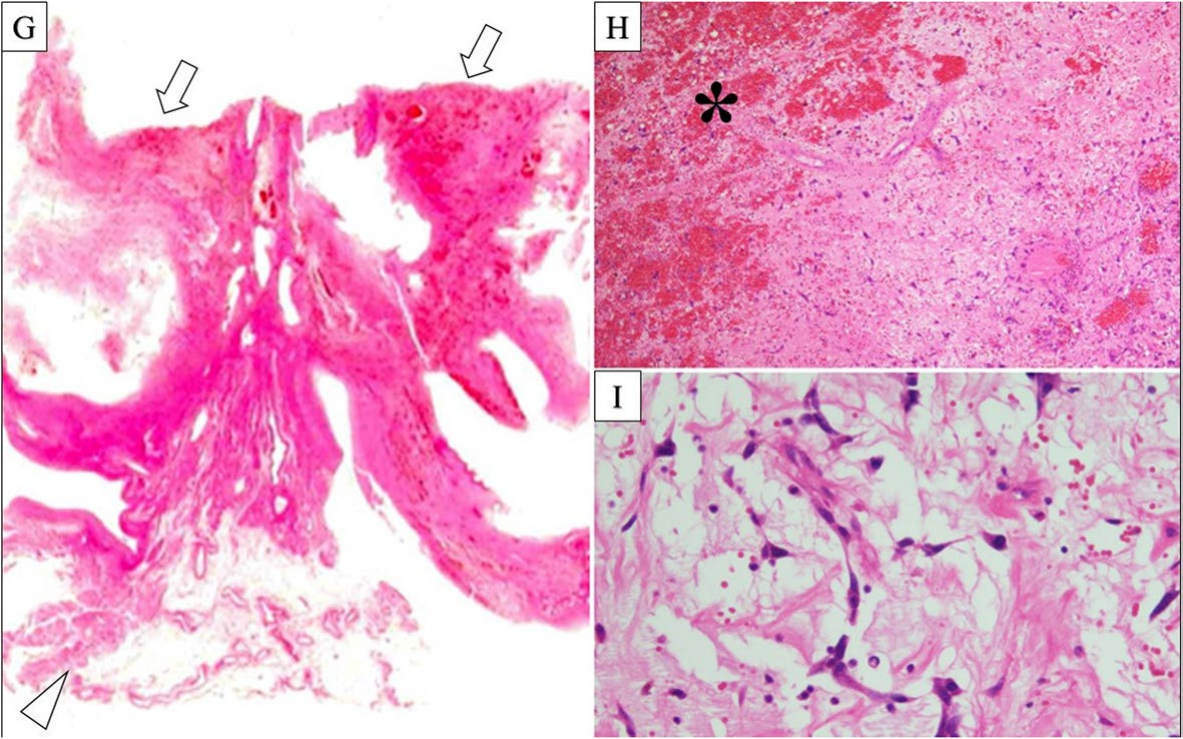
Pathological images. **G** The arrow identifies the tumor, while the arrowhead reveals the right atrium. **H** * demonstrates the intra-tumor hemorrhage. **I** The tumor cells demonstrated spindle-shape and stellate morphology with myxomatous change

Prior to the mid-sternum incision, a drainage tube was inserted under fluoroscopic guidance from the right femoral vein, positioned at inferior vena cava level. After the mid-sternum incision, the patient underwent moderate hypothermic cardiopulmonary bypass (32°C) with ascending aortic perfusion along with drainage of the superior vena cava (SVC) and right femoral vein. Upon opening the right atrium, the tumor appeared dark red, grape cluster-like, papillary, and easily disintegrated (Fig. 2). The tumor originated from the fossa ovalis and extended towards the posterior side of the right atrium, reaching the border near the left atrium. The tumor was excised, including the margins around the right atrium, left atrium, and atrial septum. The defects were reconstructed using continuous sutures with a bovine pericardial patch using 4-0 polypropylene sutures. The cardiopulmonary bypass time was 112 min and the operation time was 290 min. Pathological examination confirmed that the tumor was a myxoma with clear surgical margins. The tumor exhibited papillary growth and signs of intratumoral hemorrhage. The tumor cells exhibited spindle-shaped and stellate morphology with myxomatous changes (Fig. 3). The patient was discharged on postoperative day 11 without any complications.

## Discussion

Primary cardiac tumors are rare, with approximately 70% of cases being benign [1]. The most common benign tumor, myxoma, is observed in 75% of cases originating from the left atrium and approximately 20% arising from the right atrium. Myxoma occurs with a family history in 5–10% and is likely to have multiple and atypical locations and recur after surgery [2]. On the other hand, angiosarcomas is the most common malignant cardiac tumor, occurring predominantly in the right atrium and accounting for 90% of cases [3].

Cardiac tumors are diagnosed based on their origin or morphological features. Various imaging modalities are useful for monitoring and evaluating tumors, including transthoracic echocardiography, transesophageal echocardiography, contrast-enhanced CT, and CMR. However, surgical resection and histological examinations are required for a definitive diagnosis. In this case, the patient has no family history and no history of previous cardiac surgery. Contrast-enhanced CT revealed an irregular surface, heterogeneous contrast enhancement, and vascular structures. CMR revealed hemorrhagic necrosis with abundant blood vessels. These findings suggested the presence of a malignant tumor, specifically an angiosarcoma. However, pathological examination confirmed that the tumor was a myxoma with internal vasodilation and hemorrhage.

Catheter ablation is typically carried out using the Brockenbrough procedure, which involves puncturing the inferior portion of the fossa ovalis. In this case, laser catheter ablation has been performed with this technique. Furthermore, the myxoma originates from the same location that was punctured.

There are several possible diagnoses for a mass in the right atrium after catheter ablation. Most cases were likely thrombi or endocarditis. The causal relationship between myxoma development and catheter ablation remains poorly understood; however, several cases have been reported. These cases demonstrate myxomas growing within 2 months to 6 years after catheter ablation [4, 5]. Some of these cases exhibit rapid growth and intratumor hemorrhage, potentially related to tissue injury during the catheter ablation procedure. The shunt flow persisting in idiopathic atrial septal defect after the Brockenbrough procedure also could potentially contribute to myxoma growth. To our knowledge, there have been no other cardiac tumor types besides myxomas after catheter ablation. In addition, maze procedure and other transseptal approaches also invade the right atrium, but there have been no reports of rapidly growing tumors following them. Further investigation into the relationship between tumors and the procedure is necessary.

## Conclusion

Herein, we report a case of a giant right atrial myxoma following catheter ablation. However, the relationship between myxomas and catheter ablation requires further investigation.

## Data Availability

The datasets of this article are available on reasonable request.

## Abbreviations

CRP: C-reactive protein
CT: Computed tomography
CMR: Cardiovascular magnetic resonance imaging
SVC: Superior vena cava
